# Safety and Efficacy of Radiofrequency Catheter Ablation for Tachyarrhythmia in Children Weighing Less Than 10 kg

**DOI:** 10.1007/s00246-017-1766-7

**Published:** 2017-11-08

**Authors:** Noriyasu Ozaki, Yoshihide Nakamura, Tsugutoshi Suzuki, Jun Yoshimoto, Keiko Toyohara, Hitoo Fukuhara, Hiroshi Katayama, Kanta Kishi, Yutaka Odanaka, Hiroshi Tamai

**Affiliations:** 1 The Department of Pediatrics, Osaka Medical College, 2-7 Daigakumachi, Takatsuki, Osaka 569-0801 Japan; 2 The Department of Pediatrics, Medicine of Kinki University, Osaka, Japan; 3 The Department of Pediatric Electrophysiology, Osaka City General Hospital, Osaka, Japan; 4 The Department of Pediatric Cardiology, Shizuoka Children’s Hospital, Shizuoka, Japan; 5 The Department of Pediatric Cardiology, Tokyo Women’s Medical University, Tokyo, Japan; 6 Hitoo Fukuhara’s Kids Clinic, Japanese Red Cross Wakayama Medical Center, Wakayama, Japan

**Keywords:** Catheter ablation, Electroanatomical mapping system, Complications

## Abstract

An increasing number of children are undergoing radiofrequency catheter ablation (RFCA) for tachyarrhythmia. However, infants and toddlers undergoing RFCA are often resistant to medication or need to eliminate arrhythmia substrate, and the risks of RFCA complications are still high in infants and toddlers. From April 2008 and December 2016, 285 children who underwent radiofrequency catheter ablation (RFCA) were stratified according to body weight (group A, less than 10 kg, *n* = 22; group B, over 10 kg, *n* = 263) and the clinical features of RFCA were retrospectively reviewed in these groups. Indications for RFCA included drug-refractory tachyarrhythmia or symptomatic tachycardia and tachycardia-induced cardiomyopathy. The acute success rate in this group was 90.9%, with a relatively low recurrence rate (15.0%) after 7.0 ± 1.6 years follow-up. We performed RFCA using only 2–4 catheters in all cases. Major complications included complete right bundle branch block in one patient. No significant differences in rates of success, recurrence, or complications were noted between children weighing less and more than 10 kg. RFCA is safe and efficacious for tachyarrhythmia even in patients weighing less than 10 kg.

## Introduction

Radiofrequency catheter ablation (RFCA) is the standard treatment for tachyarrhythmia in school-aged children [1, 2], and this procedure has recently been shown to be safe and effective for application in infants and toddlers as well [3, 4]. Therefore, RFCA is being increasingly performed for children with tachyarrhythmia. Irrespective of whether they have congenital heart disease or not, infants and toddlers undergoing ablation are often resistant to medication or need substrate elimination for tachyarrhythmia.

In patients weighing less than 10 kg, the caliber of approaching vessels is relatively small, so the number of usable catheters is limited. Thus, the risk of RFCA complications may be high in these individuals. However, with advances in technology and greater experience with RFCA, the challenges of RFCA in patients less than 10 kg have been gradually overcome.

In the present study, we retrospectively reviewed cases of RFCA in children weighing less than 10 kg.

## Methods

We retrospectively reviewed the clinical features of RFCA performed in children less than 10 kg between April 2008 and December 2016. We performed RFCA at Japanese Red Cross Wakayama Medical Center, Osaka City General Hospital, and Osaka Medical College. Written informed consent was obtained from the parents of all patients and from older patients before the electrophysiology study and RFCA. Further, this study was approved by the local ethics committee.

Two hundred and eighty-five children who underwent RFCA (total 307 sessions) were stratified according to weight (group A, less than 10 kg, *n* = 22, 26 sessions; group B, over 10 kg, *n* = 263, 281 sessions). All patients were symptomatic, with tachycardia-induced cardiomyopathy or severe functional and hemodynamic deterioration caused by tachyarrhythmia. RFCA was indicated when tachyarrhythmia was refractory to anti-arrhythmic agents including amiodarone and sotalol as well as a combination of class Ic and III agents.

Electrophysiology study was performed before RFCA in all patients. After intravenous anesthesia with propofol and remifentanil, we performed intubation and artificial ventilation. We inserted 1–3 mapping catheters via the femoral and inter-jugular veins or esophagus and one ablation catheter from the femoral artery or femoral vein depending on the site of the lesion. The size of the catheters ranged from 4 to 8.5 French. Basic electrophysiological study was performed and arrhythmia inducibility was determined in each patient. We used standard atrial and ventricular burst pacing and extra stimulation. If arrhythmia could not be induced via the standard protocol, isoproterenol infusion was administered to enhance inducibility. Biplane fluoroscopy and a 3D electroanatomical mapping system (CARTO XP^®^ and CARTO 3^®^; Biosense Webster, Diamond Bar, CA) were used for localization during mapping and ablation. We used the temperature control mode with 65 °C as the highest temperature and 40 W as the highest energy. The endpoint of RFCA was non-inducibility of clinical tachycardia and equal to or less than 1-echo beat after isoproterenol infusion for reentrant tachycardia involving dual atrioventricular (AV) node pathways or twin AV nodes. After the session, all patients were administered aspirin (5 mg/kg) once daily for 1 month. After RFCA, the patients kept on staying in the hospital so that we could check if clinical tachyarrhythmia recurred or complications occurred. Thereafter, they were followed up in outpatient clinics. Acute success was defined as elimination of inducible clinical tachycardia and pre-excitation on electrophysiology examination. Complications were categorized as major or minor: the former were not resolved before discharge, while the latter were resolved during catheter laboratory.

Statistical analysis was performed using JMP version 12© (SAS institute Inc., Cary, NC, USA). The categorical variables of ablation success, complications, and patient characteristics were compared between the groups using the *χ* ^2^ test, or, when the expected value in a cell was < 5, Fisher’s exact test. Continuous variables were compared using the unpaired Student’s *t* test or Mann–Whitney *U* test.

Risk factors of complications were examined by univariate and multivariate analysis. Values of *P* < 0.05 were considered to indicate statistical significance.

## Results

Basic patient characteristics are shown in Table 1. The two groups differed significantly in terms of age, body weight, and body height. No other characteristics between two groups showed significant differences, except the incidence of congenital heart disease (45% in group A vs. 18% in group B, *P* < 0.01). The mean age at the time of RFCA in group A was 1.0 years and the mean body weight was 7.7 kg. The male/female ratio was 9/13. The arrhythmia mechanisms in both groups are also shown in Table 1. Atrioventricular reentrant tachycardia (AVRT) either through manifest or intermittent and concealed accessory pathway (AP) was found in 11 patients. Atrioventricular nodal reentrant tachycardia (AVNRT) was found in three patients. Other arrhythmia mechanisms were as follows: ectopic atrial tachycardia in two patients, ventricular tachycardia (VT) in one patient, AVRT with twin AV nodes sling in two, junctional ectopic tachycardia (JET) in 1, and multiple mechanisms in 1. Of the 22 patients in group A, ten patients (45%) had congenital heart disease (Table 1). Three patients had heterotaxy syndrome with right atrial isomerism and complex congenital heart disease (one patient had AVRT with twin AV nodes sling, another had AVNRT, and the last patient had concealed AP). Two patients had Ebstein’s anomaly: 1 patient had midseptal AP, while the other had tachyarrhythmia of multiple mechanisms, namely, manifest AP and VT. In the latter patient, the manifest AP and VT were derived from an atrialized right ventricle. The AP was eliminated in the first RFCA session, but VT could not be eliminated, and the patient required anti-arrhythmic agents.

**Table 1:** Patients characteristics and mechanisms of arrhythmia

	Group A	Group B	*P* value
	< 10 kg patients	≧ 10 kg patients	
	*n* = 22	*n* = 263	
Age (years, mean ± SD)	1.0 ± 0.6	9.6 ± 3.8	P < 0.001
Gender (M/F)	9/13	143/120	NS
Ht (cm, mean ± SD)	68.8 ± 6.6	133.8 ± 24.4	P < 0.001
Wt (kg, mean ± SD)	7.7 ± 1.5	33.7 ± 14.7	P < 0.001
Congenital heart disease (%)	10/22 (45%)	46/263 (18%)	P < 0.01
Follow-up periods (years, mean ± SD)	7.0 ± 1.6	6.5 ± 1.4	NS
Accessory pathway	11	120	
AVNRT	3	38	
Ectopic atrial tachycardia	2	19	
AVRT with twin AVN sling	2	1	
Frequent PVCs/VT	1	52	
Junctional ectopic tachycardia	1	3	
Atrial flutter	1	8	
Intra-atrial reentrant tachycardia	0	12	

*Ht* body height, *Wt* body weight, *M* male, *F* female, *SD* standard deviation, *NS* not significant, *AVNRT* atrioventricular nodal reentrant tachycardia, *AVRT* atrioventricular reentrant tachycardia, *AVN* atrioventricular node, *PVC* premature ventricular contraction, *VT* ventricular tachycardia

Both electrophysiology study and RFCA were conducted in all cases (Table 2). The total number of catheters used in group A, including mapping and ablation catheters, was less than that in group B (3.0 ± 0.8 in group A and 4.3 ± 0.7 in group B; *P* < 0.001). Additionally, the fluoroscopic time in RFCA was lower in group A than in group B (18.3 ± 9.5 min in group A and 25.5 ± 13.9 min in group B; *P* = 0.005). The total procedure time in group A was shorter than group B, although this difference was not statistically significant (147.0 ± 49.4 vs. 155.7 ± 53.8 min). Acute success was achieved in 20 of the 22 patients in group A, yielding a success rate to 90.9%. The first RFCA procedure failed in one patient with a structurally normal heart who had incessant congenital JET and severe heart failure. However, we were able to successfully treat this child in the second session. The other patient in whom the first RFCA was unsuccessful had a normal heart but multiple atrial tachycardia. We tried to eliminate the tachycardia foci, but atrial tachycardia was not induced in the first session. Therefore, this patient continued taking anti-arrhythmic agents.

**Table 2:** RFCA procedure data

	Group A	Group B	*P* value
	<10 kg patients	≧ 10 kg patients	
	*n* = 22	*n* = 263	
Number of catheter (*n*, mean ± SD)	3.0 ± 0.8	4.3 ± 0.7	P < 0.001
Fluoroscopic time (min, mean ± SD)	18.3 ± 9.5	25.5 ± 13.9	P < 0.01
Procedure time (min, mean ± SD)	147.0 ± 49.4	155.7 ± 53.8	NS
Acute success rate of 1st session (%)	20/22 (90.9%)	250/263 (95.1%)	NS
Recurrence rate (%)	3/20 (15%)	18/263 (7.2%)	NS

*RFCA* radiofrequency catheter ablation

Complication data are shown in Table 3. There was one major complication. A 5-month-old girl with congenital JET had a major complication that was RBBB at first session. The second session of RFCA was successful in this patient. There was a minor complication in group A: one patient had transient complete atrioventricular block. Minor complication was resolved before the patient left the electrophysiology laboratory. There were no procedure-related deaths in either group.

**Table 3:** Complications

	Group A	Group B	*P* value
	<10 kg patients	≧10 kg patients	
	*n* = 22	*n* = 263	
Major complications (events/all sessions, %)	1/26 (3.8)	6/281 (2.1)	NS
Right bundle branch block	1	2	
Complete atrioventricular block	0	1	
Aborted cardiac arrest	0	1	
Atrial perforation	0	1	
Laryngeal edema	0	1	
Minor complications (events/all sessions, %)	1/26 (3.8)	16/281 (5.7)	NS
Transient complete atrioventricular block	1	5	
Transient right bundle branch block	0	2	
Hematoma	0	9	

The total complication rate at the time of ablation was 7.8% (24 of 307 procedures). The univariate and multivariate analysis showed that body weight of less than 10 kg was not an independent risk factor for complications (*P* = 0.09).

## Discussion

The main findings of this retrospective study of RFCA in small children weighing less than 10 kg are as follows: (1) The acute success rate in group A was 90.9%. (2) The total number of catheters used during the RFCA procedure was less in group A than group B, and (3) the rates of success, recurrence, and complications did not differ significantly between patients weighing less and more than 10 kg.

RFCA has become the primary therapy for tachyarrhythmia, especially supraventricular tachycardia, not only in adults but also in children, with reported initial success rates up to 97.9% [1, 3–7]. Although many studies support the effectiveness and safety of RFCA [2, 8, 9], more circumspection is required before performing RFCA in children than adults. Body weight and age are considered important factors that affect the outcome of RFCA, and different intracardiac structures in congenital heart disease can complicate the procedure. In addition, although some types of arrhythmia have a favorable prognosis, recurrence is still possible [10]. To our knowledge, this study is the first to focus on RFCA in patients weighing less than 10 kg.

Although this study included more patients weighing less than 10 kg than previous reports [4–6], the rates of recurrence, transient complications, and permanent complications in the less than 10 kg group (group A) were not significantly different from those in the over 10 kg group (group B), and these results were comparable to those in previous reports [11–15]. The mean fluoroscopic time was less than that in previous reports [4, 5]. Further, the initial success rate of RFCA in group A was 90.9%, which was comparable to the 95.1% in group B.

In the present study, the mean number of catheters used was 3.0 in the group of patients weighing less than 10 kg. This was significantly less than that in the group weighing over 10 kg (4.3). The size of vessels in small children is small; therefore, the total number of catheters that could be inserted was limited. Hence, we placed an electrode catheter in the esophagus when necessary. The esophagus is located behind the atrium, and through the indwelling catheters, it was possible to obtain relatively good atrial potentials. Since no catheter was mobile, they were useful as a reference during 3D electroanatomical mapping of the atrium. Thus, we could reduce the number of catheters inserted into the heart.

The overall complication rate in the groups weighing less than 10 kg (including transient complications per sessions) was 3/26 (11.5%), and only one permanent complication occurred (3.8%); and these rates are comparable to those reported in earlier studies on RFCA in children [1, 2, 6, 16–18]. In addition, the incidence of permanent complications did not significantly differ between groups A and B. The available literature reports RFCA complications rates of 8–10% in children aged less than 1.5 years or weighing less than 15 kg [3, 14]. Thus, the complication rate in our study group weighing less than 10 kg is equivalent to those reported previously. In addition, the incidence of permanent complications in group A of the present study was 3.8%. The only procedural sequela was CRBBB in a 5-month-old girl who had congenital drug-refractory JET. No procedure-related death occurred in either group. The overall recurrence rate after RFCA in our study was 15.0% (follow-up period, 6.5 ± 1.4 years), which was similar to that in other studies [5, 9, 12, 19]. Although the presence of arrhythmic foci after RFCA is a matter of concern in small children because these could be sources of new arrhythmias [20], none of our patients had new-onset arrhythmia during follow-up.

As mentioned above, the mean fluoroscopic time in the present study was shorter than that in previous reports [1, 5]. In the present study, the fluoroscopic time in group B was significantly greater than that in group A. This could be because group B included patients with intra-atrial reentrant tachycardia and VT after postoperative complex congenital heart disease, and we needed to perform meticulous mapping and careful catheter manipulation in these patients. Similar to the findings of Blaufox et al. [14], the number of patients with tachyarrhythmia and congenital heart disease was significantly higher in group A than group B. However, since there was only a single mechanism underlying the arrhythmia in most cases, the fluoroscopic time required was not long, and the total procedure time did not differ significantly between the groups.

Although the mean fluoroscopic time (18.3 min) in the present study was shorter than that reported previously, very low fluoroscopic times of 1.7 min have been reported for cryoablation of AVNRT and AVRT using 3D electroanatomical mapping systems [21, 22]. Mah et al. also reported a fluoroscopic time of 1.1–1.5 min during ablation using electroanatomical mapping in a teenager [23]. Although it is still difficult to apply cryoablation in small children at present, pediatric electrophysiologists performing RFCA should aim to minimize the fluoroscopic time and eventually completely eliminate radiation.

Our study has some limitations. First, it is a retrospective study. Second, the number of cases included is small, although it is greater than the number in previous reports. Although the incidence of complications did not differ significantly between younger and older children, further investigation is needed to confirm our findings. Further, considering the technical aspects of manipulating catheters in small children, RFCA should be conducted in more specialized hospitals with more relevant experience, in an effort to avoid complications.

## Conclusions

RFCA is safe and efficacious for tachyarrhythmia in children weighing less than 10 kg. The present study showed that the success, complication, and recurrence rates of RFCA in this patient group are acceptable. Our findings are very important given that patients weighing less than 10 kg are at the highest risk of complications associated with RFCA.
